# Brain structural and functional outcomes in the offspring of women experiencing psychological distress during pregnancy

**DOI:** 10.1038/s41380-024-02449-0

**Published:** 2024-02-28

**Authors:** Yao Wu, Josepheen De Asis-Cruz, Catherine Limperopoulos

**Affiliations:** 1 Developing Brain Institute, Children’s National Hospital, Washington, DC 20010 USA; 2 Department of Diagnostic Imaging and Radiology, Children’s National Hospital, Washington, DC 20010 USA

**Keywords:** Neuroscience, Psychology

## Abstract

In-utero exposure to maternal psychological distress is increasingly linked with disrupted fetal and neonatal brain development and long‐term neurobehavioral dysfunction in children and adults. Elevated maternal psychological distress is associated with changes in fetal brain structure and function, including reduced hippocampal and cerebellar volumes, increased cerebral cortical gyrification and sulcal depth, decreased brain metabolites (e.g., choline and creatine levels), and disrupted functional connectivity. After birth, reduced cerebral and cerebellar gray matter volumes, increased cerebral cortical gyrification, altered amygdala and hippocampal volumes, and disturbed brain microstructure and functional connectivity have been reported in the offspring months or even years after exposure to maternal distress during pregnancy. Additionally, adverse child neurodevelopment outcomes such as cognitive, language, learning, memory, social-emotional problems, and neuropsychiatric dysfunction are being increasingly reported after prenatal exposure to maternal distress. The mechanisms by which prenatal maternal psychological distress influences early brain development include but are not limited to impaired placental function, disrupted fetal epigenetic regulation, altered microbiome and inflammation, dysregulated hypothalamic pituitary adrenal axis, altered distribution of the fetal cardiac output to the brain, and disrupted maternal sleep and appetite. This review will appraise the available literature on the brain structural and functional outcomes and neurodevelopmental outcomes in the offspring of pregnant women experiencing elevated psychological distress. In addition, it will also provide an overview of the mechanistic underpinnings of brain development changes in stress response and discuss current treatments for elevated maternal psychological distress, including pharmacotherapy (e.g., selective serotonin reuptake inhibitors) and non-pharmacotherapy (e.g., cognitive-behavior therapy). Finally, it will end with a consideration of future directions in the field.

## Introduction

Mental health disorders, including stress, anxiety, and depression, are the most common complications of pregnancy. They affect up to 15% of women in the prenatal period or first postpartum year [1, 2]. This number is even higher in women with stress-related symptoms that have not reached the severity of a mental disorder. The term psychological distress is often used to encompass issues like stress, depression, or anxiety that may fall short of meeting the criteria for a mental disorder [3]. A recent study intent on measuring prenatal maternal psychological distress in healthy, highly educated, and well-resourced women suggests that 25% of women test positive for elevated levels of anxiety and stress [4]. Similarly, nearly 1 out of every 5 women experience depressive symptoms during pregnancy and after giving birth [5, 6]. The prevalence of maternal psychosocial distress has been connected to both daily life events and environmental hardships [7, 8]. Common reasons for distress include changes in the hormones related to mood changes, dealing with discomforts of pregnancy, financial problems, worries about what to expect during birth and taking care of the baby, problems with their partner or family, medical complications during pregnancy, and managing work tasks [9].

Prenatal psychological distress is widely associated with pregnancy complications, including preeclampsia [10], spontaneous abortion [11], preterm delivery [12], lower birth weight [13], and neurodevelopmental problems in the offspring. Studies examining the effects of prenatal maternal stress exposure on brain development in the offspring have focused on newborns [14–19], children [20–27], adults [28–31], and more recently, fetuses [4, 32–35]. Importantly, exposure to prenatal maternal stress is shown to have enduring and wide-ranging consequences on brain development in the offspring, including altered regional brain volumetric growth, cortical folding, metabolism, microstructure, and functional connectivity [4, 15, 19, 23–27, 35–39]. In addition, the long-term neurodevelopmental impairments of the offspring include a spectrum of cognitive, language, social-emotional, learning and memory, and behavioral problems, as well as neuropsychiatric dysfunction [13, 24, 26, 34, 40–46]. These findings underscore the need for routine mental health surveillance for all pregnant women and targeted interventions in women with elevated psychological distress.

This paper will provide an overview of normal fetal brain development while also appraising the current literature on the brain structural, functional, and neurodevelopmental outcomes in the offspring of pregnant women experiencing elevated psychological distress. In the paper we will also review the mechanisms underlying atypical brain development in prenatal stress exposure and summarize current treatments for elevated maternal psychological distress. Lastly, we will explore future directions in the field.

### Fetal brain development in healthy pregnancies

**Fig. 1:**
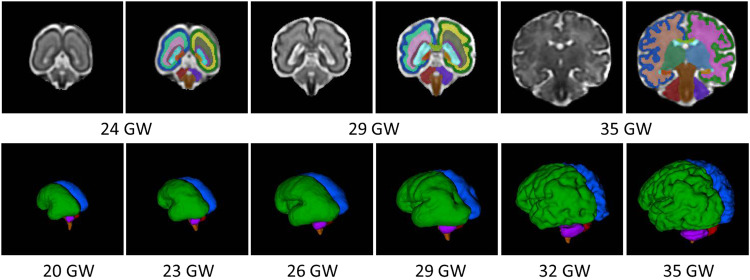
Fetal brain segmentation. Brain tissue segmentation of fetuses at 24, 29, and 35 gestational weeks (GW) (the first row); brain 3D surfaces of fetuses at 20, 23, 26, 29, 32 and 35 GW (the second row). The brain segmentation includes left (green) and right (blue) cortex, left (yellow) and right (light green) subplate, left (grass green) and right (light pink) intermediate zone, left (light purple) and right (light brown) germinal matrix, left (light orange) and right (orange) hippocampi, left (pink) and right (beige) white matter, left (light blue) and right (deep green) deep gray matter, corpus callosum (light grass green), lateral ventricle (cyan), left (purple) and right (red) cerebellum, and brainstem (brown).

**Fig. 2:**
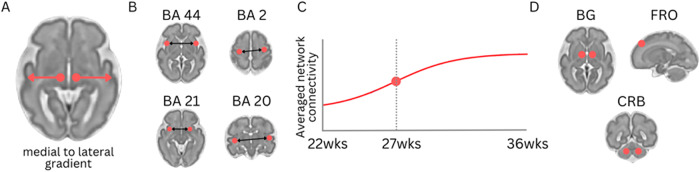
2 Fetal functional connectivity. Functional connectivity strength follows a medial to lateral developmental gradient [56, 62] (**A**). In brain regions (red dots in (**B**)) such as inferior frontal cortex (Brodmann areas, BA, 44), primary sensorimotor cortex (BA 2), middle temporal gyrus (BA 21), and inferior temporal gyrus (BA 20), connectivity strength between homologous areas increases with advancing gestational age [56]. In utero, overall brain connectivity showed a sigmoid, non-linear expansion curve, peaking between 26 and 29 weeks [adapted from [65]] (**C**). Connections arising from regions in (**D**) reliably predict biologic sex; BG basal ganglia, CRB cerebellum, and FRO frontal [68].

The human fetal brain begins to develop during the third week of gestation but grows rapidly during the prenatal period, especially in the third trimester [47]. Ultrasound is the primary modality used to assess the fetus, but its low image resolution limits detailed anatomical evaluation of the brain. With advances in ultra-fast magnetic resonance imaging (MRI) alongside the development of dedicated postprocessing tools addressing fetal motion, it is now possible to quantify global and regional tissue-specific fetal brain growth and brain function in vivo (Figs. 1 and 2). Volumetric growth of the fetal brain is reported to increase by an average of 2.3 mL per day, with fetal brain volume averaging 10% of total fetal volume throughout the third trimester in healthy fetuses [48]. During mid-gestation, the supratentorial volume, subplate, intermediate zone, and deep gray nuclei have all shown increases of around 15% per week between the 20–31 gestational weeks (GW). Likewise, the cortical plate increases by approximately 18% per week. The ventricles also grow at a more modest rate of 9.18% per week. Interestingly, the germinal matrix volume slightly increases then decreases after 25 GW [49]. The cerebellum demonstrates the greatest growth rate during mid-late gestation from 18–40 GW [50] followed by the white matter, cortical gray matter, deep subcortical structures, brainstem, and lateral ventricles [47, 50]. It is important to note that asymmetric brain growth is present prenatally, where the left cerebellar hemisphere, cortical gray matter, and deep subcortical structures have larger volumes than the right in earlier gestation. These differences, though, equalize by term, and the white matter volume is reported to be larger on the right hemisphere before 28 GW and after 36 GW [50].

In addition to volumetric measures, 3D morphometric analysis of the human fetal cerebellum shows that cerebellar growth outpaces that of the cerebrum and describes how cerebellar growth impacts the shape of the structure between 20–31 GW [51]. Specifically, transcerebellar diameter, vermal height, and vermal anterior to posterior diameter increase significantly at constant rates. Expansion along the inferior and superior aspects of the cerebellar hemispheres results in decreased convexity along the inferior vermis and increased convexity of the medial hemisphere representing development of the paravermian fissure [51]. Another study on shape analysis of the brainstem and cerebellum compares healthy fetuses between 30–40 GW with age-matched ex-utero premature infants [52] and suggests that the left and right cerebellar hemispheres grow faster compared to the vermis, and the pons grows faster than the midbrain and medulla in both groups [52].

Cortical surface analyses and gyrification indices are also used to characterize fetal cerebral cortical development [53, 54]. A study of healthy fetuses at 25–35 GW shows an exuberant third-trimester gyrification process and suggests a non-linear evolution of sulcal development [53]. Another study of fetuses at 21.7–38.9 GW indicates that after a slow initial start, cortical folding increases rapidly between weeks 25–30. Folding subsequently slows down closer to birth. The same study also analyzes regional patterns in folding by parcellating the fetal cortex using a nine-region anatomical atlas. The results show regional differences in growth rate, with the parietal and posterior temporal lobes exhibiting the fastest growth. Additionally, the cingulate, frontal and medial temporal lobes also develop, but at a slower rate [55]. Taken together, these in-vivo studies of the fetal brain using quantitative MRI confirm the robust brain growth that takes place in utero.

Apart from mapping trajectories of structural brain growth in utero, MRI has also enabled in-vivo evaluation of fetal brain functional connectivity [56, 57]. In fetuses and newborns, resting-state fMRI (rs-fMRI) is the predominant technique for imaging emerging brain networks for its ability to interrogate multiple systems simultaneously with minimal demands on the participant. Resting-state fMRI measures blood oxygenation level-dependent (BOLD) signal changes; brain activity is inferred, in turn, from the BOLD response. In 2012, early in utero rs-fMRI studies detected occipital and frontal networks in the developing brain [58, 59]. Since then, advances in image preprocessing and analysis have enabled more comprehensive evaluations of the fetal brain [60, 61]. Akin to structural maturation, regional differences in functional connectivity trajectories have also been observed in utero. Consistent with axonal growth patterns, a medial-to-lateral gradient of network organization has been demonstrated, such that connections between homologous medial structures are stronger than those connecting lateral areas in utero. Connectivity strength between most symmetric regions has been shown to increase with advancing gestational age [56, 62]. Related to this, one recent study has suggested that select networks track brain maturity. Specifically, a network resembling the global signal in adults has been shown to reliably predict the gestational age [63]. The relationship between connectivity and age, however, is neither always positive nor linear. Posterior cingulate connectivity to the rest of the brain, for example, weakens with increasing gestational age [64]. Likewise, network strength shows a non-linear, sigmoid expansion mid-gestation first at the occipital lobe at around 26 GW, followed by the temporal, frontal, and parietal networks [65]. Notably, non-linear components of networks tend to predict fetal age more accurately than conventional linear models [66]. Associations between gestational age and connectivity also varies with sex, with male-female differences seen in the posterior cingulate-temporal, fronto-cerebellar, and intracerebellar connections [67]. Connections involving the somatomotor regions, frontal cortices, and basal ganglia have also been shown to reliably predict biologic sex [68].

Beyond individual connections, systemic network approaches have also provided researchers with a powerful tool to concisely map fetal functional brain organization. Fetal networks, like adults, exhibit efficient small-world organization, suggesting that regions are simultaneously well integrated with topologically distant regions of the brain while forming specialized clusters with their close-by neighbors [69, 70]. Fetal resting-state networks also tend to form clusters or modules; this tendency, called modularity, decreases with advancing gestational age [69, 70]. Using this analytic framework, regions critical to brain network integrity, called hubs, have also been identified. Most hubs are localized in the cerebellum, while some are in the primary and association cortices [71].

In contrast to rs-fMRI, task-based experiments are designed to elicit sensory-driven brain responses, thus, activating targeted networks. Because of the demands on the subject, this setup is often not ideal for fetuses. Even so, there have been a few in-utero task-based studies [72–76]. Most of these examine fetal responses to auditory stimulus (e.g., maternal voice and music) and show activation in audition-related regions in the temporal lobe, including the Heschl’s gyrus. Although these studies are limited by a small sample size, they suggest the potential of directly exploring emerging sensory processes in the fetal brain.

Altogether, MRI studies have provided unprecedented insights into fetal brain development. However, several issues related to both the technical challenges of in-vivo fetal MR imaging and the rapidly evolving anatomy of the developing brain need to be considered when planning and interpreting fetal MRI studies. Motion correction remains challenging as fetuses move in a relatively unconstrained manner, although advances in fetal MRI methodologies [77–79] have helped reduce the impact of high motion on MR images. Further, some brain regions, including those that play a role in stress such as the amygdala and hippocampus (discussed below), may be difficult to reliably differentiate on fetal MRI due to their small size and the minimal contrast between these regions in the fetal period; this is an issue that could be further compounded by motion. Studies have suggested combining the image intensity information with anatomical features to segment the fetal hippocampus on structural MRI [80, 81]. However, accurate segmentation of the fetal amygdala is an unsolved challenge. For functional MRI, similar to adults, the neurobiology of the fetal BOLD response is not well understood. Further investigation is needed to determine whether hemodynamic responses in fetuses also arise from postsynaptic local field potentials [82, 83], as suggested by evidence in adults. Nevertheless, with all the ongoing changes in the developing brain (e.g., angiogenesis, neurogenesis, synaptic formation, etc.), significant differences between the adult and fetal BOLD response may be equally as likely [84].

In summary, these structural and functional studies describing normal in-vivo brain development with the use of safe and non-invasive imaging techniques have provided critical insights into the progression of in utero fetal brain development, and have provided an important tool for measuring alterations in fetal brain development associated with maternal stress exposure, facilitating earlier identification and targeted early intervention [47, 55].

### Brain development outcomes in the offspring of pregnant women who experienced elevated maternal psychological distress

#### Prenatal maternal psychological distress and brain structural development

Intra-uterine exposure to maternal psychological distress has been linked with early and long-term alterations to brain development in the offspring (Table 1). Elevated maternal psychological distress during mid-gestation is associated with a decrease in the newborn’s head circumference [13], a decrease in the regional cerebrum and cerebellum gray matter volumes of children at 6–9 years of age [23], a reduction in cortical thickness in the bilateral precentral gyrus and dorsolateral prefrontal cortex in newborns [18], the right inferior frontal and middle temporal regions at 2–5 years old [25], the frontal and temporal regions in children at 7 years old [85], and the whole cortex and frontal lobes in children at 6–9 years old [21, 24]. Interestingly, prenatal maternal stress is also associated with decreased cortical gray matter volume and increased cortical gyrification in adult offspring [28, 30, 31].

**Table 1:** The impact of prenatal maternal psychological distress on human brain and behavioral development in the offspring.

Article	Subject size	Maternal distress measure	Imaging modality	Age at scan	Child behavior measure	Age at behavioral testing	Results
Wu et al. [4]	119	STAI; PSS; EPDS at 24–40 GW	sMRI; MRS	24–40 GW	–	–	Maternal trait anxiety was associated with smaller fetal left HIP volume. Maternal anxiety and stress were associated with increased fetal cortical gyrification in the frontal lobe and temporal lobe. Elevated maternal depression was associated with decreased creatine and choline levels in the fetal brain.
De Asis-Cruz et al. [33]	50	STAI, PSS, EPDS at 24–39 GW	rs-fcMRI	24–39 GW	–	–	Region specific changes in connectivity associated with trait and state anxiety, for example, FC between the superior frontal regions and somatosensory cortices correlated positively with trait and state anxiety scores.
Pradhan et al. [103]	131	PSS, STAI at 18–37 GW	MRS	18–37 GW	–	–	Eleveted maternal stress and anxiety were associated with higher levels of lactate in the fetal brain. Higher levels of lactate and scyllo-inositol in fetuses from the pandemic cohort vs pre-COVID pandemic.
Van Den Heuvel et al. [106]	64	CES-D, STAI, PSWQ, PSS, SWLS at MRI visit	rs-fcMRI	20–39 GW	subscale of CBC, actigraphy data analyses	3 and 5 yrs	Increased maternal stress and negative affect were associated with increased sleep problems during toddlerhood and weakened fetal CRB-insular FC; altered fetal FC did not mediate association between maternal negative affect and toddler sleep problems.
Thomason et al. [201]	118	CES-D, STAI-T, PSWQ, PSS, SWLS	rs-fcMRI	26–39 GW	–	–	Increased maternal prenatal negative affect/stress was associated with alterations in fetal frontoparietal, striatal, and temporoparietal connectivity.
Rajagopalan et al. [91]	45	Coronavirus Perinatal Experiences-Impact Survey; Brief Coping Orientation to Problems Exposed questionnaire	sMRI; rs-fcMRI	24–38 GW	–	–	Increased maternal perception of pandemic-related stress was associated with increased normalized fetal brainstem volume and reduced global fetal brain temporal functional variance.
Hendrix et al. [107]	77	PSS, CES-D, STAI; maternal cortisol at MRI visit	rs-fcMRI	32.82±3.86 GW	–	–	Maternal distress positively correlated with HIP connectivity to the right posterior parietal cortex, this relationship was moderated by fetal sex; higher maternal cortisol associated with increased coupling between HIP and dorsal anterior cingulate cortex, left medial PFC.
Wu et al. [35]	140	STAI; PSS; EPDS at 21–40 GW	sMRI	21–40 GW	–	–	Maternal stress and anxiety were associated with smaller left and right HIP and CRB volumes among women with fetal congenital heart disease. Impaired HIP regions were noted in the medial aspect of left HIP head and inferior aspect of right HIP head and body. Impaired CRB regions were noted in the anterior superior aspect of vermal and paravermal regions and the left CRB lobe.
Wu et al. [34]	97	STAI; PSS; EPDS at 24–40 GW	sMRI; MRS	24–40 GW	Bayley-III; ITSEA; PSI-SF	18 mos	Prenatal maternal stress was negatively associated with infant cognitive performance, and this association was mediated by fetal left HIP volume.
Lu et al. [32]	202	STAI; PSS; EPDS at MRI visit	sMRI	26.1–35.3 GW	–	–	Elevated maternal anxiety and stress were associated with smaller fetal HIP and CRB volumes. Higher trait anxiety was associated with lower white matter volume. Elevated maternal anxiety and depression were associated with higher cortical sulcal depth.
Ramphal et al. [122]	112	Composite SES score from various sources measured at birth	rs-fcMRI	39.3 ± 1.2 wks	ITSEA	2 yrs	Low SES at birth, which has been linked to maternal stress has also been correlated with altered neonatal striatal and medial prefrontal connectivity, which in turn mediated the relationship with low socio-economic status and behavioral inhibition at two years of age
Moog et al. [14]	86	PSS in early, mid and late pregnancy	sMRI	5–64 days	Bayley-III	6 and 12 mos	Maternal PSS was inversely associated with newborn left HIP volume. Newborn left HIP volume was positively associated with infant social-emotional development across the first year after birth.
Lautarescu et al. [92]	413	EPDS at prenatal 22–40 GW and postnatal 36.57–44.71 GW	DTI	36.57–44.71 weeks PMA	CBC; Q-CHAT; BSID-III; Parenting Scale	18 mos	Maternal depression was positively associated with infant fiber density in the uncinate fasciculus, with left uncinate fasciculus fiber density, in turn, positively associated with social-emotional abilities in toddlerhood. Higher maternal depression predicted toddler social-emotional difficulties, but this relationship was not mediated by fiber density in the left uncinate fasciculus.
Groenewold et al. [86]	124	BDI at 28–32 GW	sMRI	2–6 weeks after birth	–	–	Larger volumes in the right AMY and bilateral caudate nucleus were observed in prenatal maternal depression exposed compared to unexposed infants. Larger HIP volume in prenatal maternal depression exposed females only.
Qiu et al. [36]	175	STAI at 26 GW	sMRI	At birth; 6 mos	–	–	No influence of maternal anxiety on HIP volumes at birth. Neonates with maternal anxiety showed slower growth of bilateral HIP volumes over first 6 months compared to neonates with no antenatal maternal anxiety.
Qiu et al. [18]	146	STAI at 26 GW	sMRI	Newborn	–	–	Negative associations of maternal anxiety with cortical thickness in bilateral precentral gyrus and the dorsolateral PFC. Positive associations of maternal anxiety with cortical thickness in the right ventrolateral PFC and the right superior parietal cortex and precuneus.
Lautarescu et al. [202]	251	STAI; SLE in the past yr before study	DTI	37.86–45.71 GW	–	–	Higher SLE scores were associated with higher axial, radial, and mean diffusivity in the left uncinate fasciculus, and higher axial diffusivity in the right uncinate fasciculus in preterm infants at term age.
Graham et al. [93]	34	STAI; BDI during late pregnancy	DTI	2 wks after birth	–	–	Maternal STAI scores negatively correlated with FA values in the left orbitofrontal, prefrontal, and middle frontal WM, and the right middle frontal WM; BDI scores negatively correlated with FA values in one cluster in right middle frontal WM.
Rifkin-Graboi et al. [15]	157	EPDS at 26 GW	sMRI; DTI	6–14 days	–	–	Lower FA and axial diffusivity but not volume in the right AMY in the infants of mothers with high vs. those with low-normal EPDS scores.
Rifkin-Graboi et al. [16]	54	STAI at 26 GW	DTI	5–17 days	–	–	Elevated maternal anxiety was associated with reduced FA in right insular cortex, middle occipital and inferior temporal regions, angular gyrus, uncinate fasciculus, posterior cingulate, parahippocampus, dorsolateral prefrontal, inferior frontal regions, and inferior fronto-occipital fasciculus, and bilateral superior temporal and left postcentral regions.
Lehtola et al. [89]	123	SCL-90 and EPDS at 14, 24 and 34 GW	sMRI	2–5 wks	–	–	Prenatal maternal SCL + EPDS sum score was negatively associated with newborn left and right AMY volumes in males only.
Acosta et al. [87]	105	EPDS at 14, 24 and 34 GW	sMRI	11–54 days	–	–	EPDS scores were weakly positively associated with right AMY volume in infants with a low polygenic risk score for major depressive disorder (PRS-MDD) and weakly negatively in infants with a high PRS-MDD. EPDS at 24 GW showed a sex-specific interaction with PRS-MDD.
Hashempour et al. [17]	84	EPDS at 14, 24 and 34 GW	DTI	11–54 days	–	–	Compared with girls, boys exposed to greater maternal depressive symptoms during 14 GW showed higher left AMY MD.
Dean et al. [94]	101	EPDS, STAI at 28 and 35 GW	DWI	18–50 days	–	–	Elevated maternal depression and anxiety were associated with decreased neurite density and increased mean, radial, and axial diffusivity in right frontal white matter microstructure. Lower FA and intracellular volume fraction in females and higher FA and intracellular volume fraction in males exposed to elevated maternal depression and anxiety.
Scheinost et al. [19]	26	Retrospective review for diagnosis of depression and/or anxiety in the maternal medical chart	sMRI; rs-fcMRI	35–40 wks PMA	–	–	Preterm neonates with exposure to prenatal stress show less connectivity between the left AMY and the thalamus, the hypothalamus, and the peristriate cortex. Exposure to prenatal stress exacerbates reductions in limbic connectivity in very premature newborns, such that AMY and subcortical FC was reduced in stress-exposed vs non-exposed preterm infants.
Scheinost et al. [108]	46	PSS, RADS, PDQ during the 2nd and 3rd trimesters	rs-fcMRI	40–44 wks PMA	mobile conjugate reinforcement task	4 mos	Maternal distress during the 3rd trimester was associated with weaker HIP-cingulate cortex and HIP-temporal lobe FC in newborns. 2nd trimester cortisol levels were linked to reduced HIP-cingulate FC and increased HIP-temporal FC; weaker bilateral HIP and posterior cingulate cortex connectivity linked to higher maternal depression; connectivity between the HIP and dorsal anterior cingulate cortex, which was inversely associated with maternal stress, was also noted to be positively correlated with infant memory.
Rajasilta et al. [112]	21	EPDS, anxiety subscale of SCL-90 at 24 GW	rs-fcMRI	42.43–46.43 wks PMA	–	–	Composite depression and anxiety scores positively associated with the amplitude of regional neuronal activity in the newborn medial PFC.
Brady et al. [110]	319	SES and crime data from various sources	rs-fcMRI	28–41 wks PMA	–	–	Reduced AMY and anterior DMN connectivity in newborn offspring of women exposed to high prenatal psychosocial stress and who are living in neighborhoods with high property or violent crimes.
Graham et al. [117]	70	cortisol 5 times/day for 4 consecutive days in early, mid, and late gestation; CES-D over the first 2 years of life	rs-fcMRI	3.65 ± 1.72 wks	CBC	24 mos	Higher cortisol levels correlated with greater AMY connectivity to networks like the DMN and emotion regulation circuitry; AMY connections that are strongly correlated with maternal cortisol levels predicted internalizing scores on the CBC at 2 years of age; this effect of cortisol on outcomes was observed in females only.
Posner et al. [111]	64	PSS, HAM-D, CES-D at 34–37 GW	rs-fcMRI	5.8 ± 1.7 wks	–	–	Heightened depression during the 3rd trimester were associated with reduced connectivity to PFC circuits around 5 weeks of life.
Manning et al. [113]	75	EPDS, PROMIS Anxiety scale, SSEQ prenatally and 3 mos postpartum	DTI; rs-fcMRI	92 ± 14 days	–	–	Prenatal maternal distress was associated with higher FA in the right uncinate fasciculus and lower MD in the right amygdala prefrontal white matter tract. In infants of women who were pregnant during the COVID-19 pandemic and who had low social support, there was a weaker connectivity between the right AMY and superior orbitofrontal cortex.
Humphreys et al. [109]	33	CRISYS, LSCR at prenatal visit	rs-fcMRI	4.81±0.93 months	–	–	Reduced functional coupling between AMY and mPFC in stress-exposed infants, in contrast to structural connectivity between these two areas that increased with maternal stress.
Qiu et al. [37]	24	EPDS at 26 GW and 3 mos postpartum	rs-fcMRI	66.58 ± 1.82 PMA	–	–	Elevated maternal depression in the 2nd trimester linked to increased AMY connectivity to areas of the brain involved in socio-emotional processing and memory at 6 months.
Lebel et al. [25]	52	EPDS in each trimester of pregnancy and at 3 mos postpartum	sMRI; DTI	2.6–5.1 yrs	–	–	Maternal 2nd trimester EPDS scores negatively correlated with children’s cortical thickness in right inferior frontal and middle temporal regions and with radial and mean diffusivity in lateral portions of the uncinate, inferior fronto-occipital, and arcuate fasciculi. Postpartum EPDS scores negatively correlated with children’s right superior frontal cortical thickness and with diffusivity in white matter originating from that region.
Hay et al. [95]	54	EPDS at 11, 16.8 and 31.5 GW and 3 mos postpartum	DTI	2.85–6 yrs	CBC	Within 6 mos of the MRI	3rd trimester EPDS scores were associated with higher MD in the AMY-frontal tract and the cingulum. Altered structural connectivity between the AMY and frontal cortex mediated the relationship between 3rd trimester maternal depression and child externalizing behavior in males only.
Wen et al. [39]	235	EPDS at 26 GW and 3 mos postpartum; Beck’s Depression Inventory-II at 1, 2, 3 and 4.5 yrs	sMRI; DTI	4.5 yrs	–	–	Greater prenatal maternal depressive symptoms were associated with larger right AMY volume in girls only. Increased postnatal maternal depressive symptoms were associated with higher right AMY FA in the overall sample and girls, but not in boys.
Acosta et al. [90]	28	EPDS at 14, 24 and 34 GW, 3, 6 mos and 1, 2, and 4 yrs postpartum. SCL-90-Revised at 14, 24 and 34 GW, 3, 6 mos and 2, 4 yrs. PRAQ-R2 at 24 and 34 GW	sMRI	4 yrs	–	–	Elevated depressive symptoms of the early 2nd trimester, after controlling for prenatal maternal general anxiety, were related to smaller right AMY volumes in the overall sample. Higher depressive symptoms of the 3rd trimester were associated with smaller right AMY volumes in boys compared to girls.
Acosta et al. [22]	27	PRAQ-R2 at 24 and 34 GW; EPDS and SCL-90-Revised at 14, 24, and 34 GW	sMRI	4 yrs	Strength and Difficulties Questionnaire	4 yrs	Higher pregnancy-related anxiety in the 2nd trimester was related to greater left-relative AMY volume in girls vs. boys. Both maternal pregnancy-related anxiety and child’s AMY volume are related to child emotional and behavioral difficulties. The left AMY volume may partly mediate sex-specific associations between pregnancy-related anxiety and child behavioral difficulties.
Soe et al. [114]	128	EPDS, BDI-III at 26 GW, 3 mos, 1, 2, 3, and 4.5 yrs after birth	rs-fcMRI	4.4–4.8 yrs	–	–	Greater maternal depression associated with weakened AMY connectivity of the AMY to the cortico-striatal circuitry, particularly in the insula, putamen, orbitofrontal cortex, and temporal pole in young girls.
Donnici et al. [115]	54	EPDS, SCL-90-Revised during the 2nd trimester and 12 wks postpartum	rs-fcMRI; sMRI	3–7 yrs	–	–	Elevated maternal anxiety in the 2nd trimester linked to greater negative AMY connectivity to bilateral somatosensory cortices and left inferior parietal lobule.
van der Knaap et al. [116]	39	BSI at 20–25 GW and 3 yrs after birth	Task-based fMRI	6–9 yrs	–	–	Increased AMY responsivity to pictures of negative emotional faces in children exposed to maternal depression in utero.
Buss et al. [23]	35	Pregnancy anxiety scale at mean 19, 25, and 31 GW; PSS at 8 wks after birth	sMRI	6–9 yrs	–	–	Maternal anxiety at 19 GW was associated with gray matter volume reductions in the PFC, the premotor cortex, the medial temporal lobe, the lateral temporal cortex, the postcentral gyrus as well as the CRB extending to the middle occipital gyrus and the fusiform gyrus.
El Marroun et al. [20]	636	BSI at 20.6 GW and 3 yrs.	DTI	6–9 yrs	–	–	Elevated prenatal maternal depressive symptoms were associated with higher MD in the uncinate fasciculus, and lower FA and higher MD in the cingulum bundle.
El Marroun et al. [21]	654	BSI at 20.6 GW and 3 yrs.	sMRI	6–10 yrs	–	–	Elevated prenatal maternal depressive symptoms were associated with a thinner superior frontal cortex and larger caudal middle frontal area in the left hemisphere.
Buss et al. [26]	65	Maternal cortisol at 15, 19, 25, 31, and 37 GW	sMRI	7 yrs	CBC	7 yrs	Higher cortisol levels at 15 GW were associated with larger right AMY volume and more affective problems in girls. The association between maternal cortisol levels and children’s affective problems was partially mediated by AMY volume.
Davis et al. [85]	74	PSS at 15, 19, 31, and 37 GW and 2 mos and 12 yrs after birth; CES-D at 12 yrs	sMRI	7 yrs	Children’s Depression Inventory	12 yrs	Prenatal maternal stress was associated with less cortical thickness primarily in frontal and temporal regions and with elevated depressive symptoms; child cortical thickness additionally correlated with adolescent depressive symptoms.
Davis et al. [203]	91	Plasma cortisol, STAI, and CES-D at mean 19 and 31 GW; DBI at MRI visit	sMRI	6–9 yrs	WISC-IV; Expressive Vocabulary Test-2	6–9 yrs	Elevated maternal cortisol levels during the 3rd trimester were associated with greater child cortical thickness primarily in frontal regions and enhanced child cognitive performance.
Sarkar et al. [27]	22	SLE in pregnancy and since birth; EPDS at 17 months	DTI	6–9 yrs	–	–	Maternal SLE was correlated positively with right uncinate fasciculus FA, and negatively with right uncinate fasciculus perpendicular diffusivity in children.
Sandman et al. [24]	81	CES-D at 19, 25, and 31 GW	sMRI	6–9 yrs	CBC	6–9 yrs	Maternal depression at 25 GW was associated with cortical thinning, primarily in the right superior, medial orbital, and frontal pole regions of the PFC. The association between maternal depression at 25 GW and child externalizing behavior was mediated by cortical thinning in right prefrontal areas.
Sandman et al. [204]	97	Maternal plasma measuring placenta corticotropin-releasing hormone at 15, 19, 25, 31, and 36 GW	sMRI	6–9 yrs	Child Behavior Checklist	6–9 yrs	Fetal exposure to elevated levels of placenta stress hormone (corticotropin-releasing hormone) was associated with cortical thinning, primary in temporal, paracentral, and frontal areas. At 19 GW, this association was stronger in girls vs. boys; at 31 GW, this association was globally in boys but locally in the temporal pole in girls. Reduced regional cortical volumes contribute to cognitive and emotional deficits in children.
Zou et al. [205]	3469	BSI at prenatal, 2 mos, 3 and 10 yrs postpartum; EPDS at 2 mos	sMRI; DTI	10 yrs	Brief Problem Monitor	10 yrs	Children exposed to elevated maternal depression across the perinatal period had smaller gray and white matter volumes and lower white matter FA vs. non-exposed children. The gray matter volume mediated the association between postnatal maternal depressive symptoms and child attention problems.
Jones et al. [88]	63	Storm32; IES-R; interview questions	sMRI	11.5 yrs	CBC	11.5 yrs	In boys, subjective distress during late pregnancy was associated with larger right AMY/total brain volume, which explained higher levels of externalizing behavior. In girls, later gestational exposure to the ice storm was associated with larger AMY/total brain volume; higher levels of objective prenatal stress were associated with more externalizing problems, which was mediated by larger AMY/total brain volume.
McKee et al. [38]	127	IES-R; Objective Ice Storm Hardship and Cognitive Appraisal Questionnaires	sMRI	11.5 yrs	–	–	Prenatal maternal stress exposure led to altered bilateral HIP volumes, and volumetric changes in subfields CA1, subiculum and stratum radiatum/lacunosum-moleculare. A negative maternal cognitive appraisal of the storm’s consequences predicted smaller adolescent HIP volumes.
Jensen et al. [96]	393	SLE at 18 GW, and 8, 21, 33, 47 mos, 12–16 yrs after birth	DTI; sMRI	18–21 yrs men	–	–	Prenatal stress is associated with lower magnetization transfer ratio and myelin water fraction in the genu and/or splenium of the corpus callosum, and with lower magnetization transfer ratio in white matter.
Mareckova et al. [31]	93	SLE during the first 20 GW	sMRI	23–24 yrs	Profile of Mood States questionnaire	23–24 yrs	Higher prenatal stress predicted more mood dysregulation, lower overall gray matter volume, and lower gray matter volume in mid-dorsolateral frontal cortex, anterior cingulate cortex, and precuneus in young adulthood.
Mareckova et al. [30]	85	SLE at 20 GW, at birth, and 6, 18 mos after birth	sMRI	23–24 yrs	Profile of Mood States questionnaire	23–24 yrs	Early prenatal stress was associated with sex-dependent medium-to-large effects in cortical gyrification in large temporal, parietal, and occipital regions; Later perinatal stress was associated with sex-independent small-to-medium effects in smaller, more anterior regions. In females, early prenatal stress predicted higher cortical gyrification index in a large temporal region, which was further associated with mood disturbance in adulthood.
Mareckova et al. [29]	131	Depression symptom questionnaire in midpregnancy, after birth, 6 and 18 mos after birth	sMRI	23–24 yrs	Profile of Mood States questionnaire; STAI	23–24 yrs	Elevated prenatal maternal depression showed a linear relationship with elevated brain age gap (i.e., the difference between chronological and structural brain age). Brain age gap further showed a quadratic relationship with anxiety and dysregulated mood in the adult offspring.
Mareckova et al. [206]	260	EPDS at 20 GW, 2 wks, 6 and 18 mos after birth	sMRI	23–24 yrs, repeat at 28, 30 yrs	–	–	Elevated prenatal maternal depression was associated with greater positive differences between structural and chronological brain age in adult offspring at late 20 s.
Turk et al. [207]	49	Dutch version of the STAI at 12–22 GW	rs-fcMRI	28 yrs	–	–	Exposure to high anxiety in utero linked to weakened connectivity between the medial PFC and inferior gyrus, left lateral PFC, and sensorimotor cortex.
Favaro et al. [28]	35	Interview to recall stressful events during pregnancy; Hopkins-SCL; STAI	sMRI; rs-fcMRI	14–40 yrs	–	–	Elevated prenatal stress was associated with decreased gray matter volume in the left medial temporal lobe and AMY and increased functional connectivity between the left medial temporal lobe and the pregenual cortex.

*GW* gestational week, *sMRI* structural MRI, *MRS* magnetic resonance spectroscopy, *rs-fcMRI* resting-state-functional MRI, *DTI* diffusion tensor imaging, *DWI* diffusion-weighted imaging, *PMA* post menstrual age, *CES-D* Centers for Epidemiologic Studies Depression scale, *EPDS* Edinburgh Postnatal Depression Scale, *PSS* Perceived Stress Scale, *STAI* Spielberger State-Trait Anxiety Inventory, *BDI* Beck Depression Inventory, *SLE* Stressful Life Events, *IES-R* Impact of Event Scale-Revised, *PSWQ* Penn State Worry Questionnaire, *SWLS* Satisfaction with Life Scale, *RADS* Reynolds Adolescent Depression Scale, *PDQ* Pregnancy Distress Questionnaire, *CRISYS* Crisis in Family Systems-Revised, *LSCR* Life Stressor Checklist-Revised, *HAM-D* Hamilton Rating Scale for Depression, *PROMIS* Patient-Reported Outcomes Measurement Information System, *SSEQ* Social Support Effectiveness Questionnaire, *SCL* Symptom Checklist, *BSI* Brief Symptoms Inventory, *CBC* Child Behavior Checklist, *SES* socio-economic status, *Bayley-III* Bayley Scales of Infant and Toddler Development-Third Edition, *ITSEA* Infant-Toddler Social Emotional Assessment, *PSI-SF* Parenting Stress Index-Short Form, *PRAQ-R2* Pregnancy-Related Anxiety Questionnaire-Revised 2, *Q-CHAT* Quantitative Checklist for Autism in Toddlers, *WISC* Wechsler Intelligence Scale for Children, *FC* functional connectivity, *HIP* hippocampus, *AMY* amygdala, *CRB* cerebellum, *PFC* prefrontal cortex, *FA* fractional anisotropy, *MD* mean diffusivity, *DMN* default mode network.

In addition to the cortical area, the amygdala and hippocampus are particularly vulnerable to prenatal psychological distress. Greater prenatal maternal depressive symptoms are associated with larger right amygdala volume in infants under 2 months old and girls at 4.5 years old [39, 86, 87]. Consistently, higher maternal cortisol levels in early gestation also lead to a larger right amygdala volume in girls at 7 years old [26]. Similarly, disaster-related prenatal maternal stress is associated with larger amygdala volumes in children at 11 years old [88]. On the contrary, prenatal maternal psychological problems and depressive symptoms are negatively associated with amygdala volumes in newborns and young children, especially in males [89, 90]. In the hippocampus, elevated prenatal maternal anxiety is associated with slower growth of the left and right hippocampus during the first 6 months of life [36]. A negative maternal cognitive appraisal of the 1998 Quebec ice storm’s consequences is associated with smaller hippocampal volumes in children at 11 years old [38]. While prenatal maternal depression is positively associated with the hippocampal volume in female infants at 2–6 weeks old [86]. Recent fetal studies find that elevated maternal psychological distress is associated with a decrease in fetal hippocampal, cerebellar, and white matter volumes and increases in fetal brainstem volume, cortical gyrification, and sulcal depth [4, 32, 35, 91]. These data underscore the striking changes in brain structure that ensue in the weeks, months, years, and decades after offspring are exposed to maternal psychological distress during pregnancy.

#### Prenatal maternal psychological distress and brain microstructural development

Altered white matter microstructures after prenatal stress exposure are also reported in the newborn, where maternal depression is positively associated with fiber density in the neonatal uncinate fasciculus [92]. Maternal anxiety is negatively correlated with fractional anisotropy (FA) in the neonatal right insular cortex, middle occipital and inferior temporal regions, angular gyrus, uncinate fasciculus, posterior cingulate, parahippocampus, dorsolateral prefrontal, inferior frontal regions, and inferior fronto-occipital fasciculus, and bilateral superior temporal and left postcentral, orbitofrontal, prefrontal and middle frontal gyrus regions [16, 93]. Maternal depression is also connected with lower FA and axial diffusivity in the right amygdala of newborns [15]. Compared with females, male offspring exposed to greater maternal depressive symptoms at 14 GW show higher left amygdala mean diffusivity (MD) [17]. Additionally, elevated maternal depression and anxiety are associated with decreased neurite density and increased mean, radial, and axial diffusivity in the right frontal white matter microstructure in infants [94]. In children, elevated prenatal maternal depression also correlates with lower radial and mean diffusivity in the lateral portions of the uncinate, the inferior fronto-occipital, and the arcuate fasciculi. It is also associated with higher MD in the cingulum, amygdala-frontal tract, and uncinate fasciculus, and lower FA in the cingulum [20, 25, 95]. Moreover, prenatal maternal stressful life events are positively correlated with right uncinate fasciculus FA, and negatively with right uncinate fasciculus perpendicular diffusivity in children [27]. In adult offspring, prenatal maternal stress is associated with lower magnetization transfer ratio and myelin water fraction in the genu and splenium of the corpus callosum, and lower magnetization transfer ratio in white matter in young adults [96].

#### Prenatal maternal psychological distress and brain biochemistry

Disturbances in important brain biochemicals in the setting of maternal psychological distress have also been reported, mostly in animal studies. These include reductions in N-acetylaspartate (a marker of neuronal integrity) in the frontal cortex and hypothalamus in early-life stress-exposed mice [97–99] and altered neurotransmitter metabolism of gamma-aminobutyric acid and glutamate in the right hippocampus of pre-gestational stress-exposed rat offspring [100]. A decrease in choline and creatine levels is also found in the left hippocampus and centrum semiovale in human adults with anxiety disorder [101, 102]. A recent human fetal study reports that prenatal maternal depression has a negative association with both creatine and choline levels in the fetal brain [4]. Fetal brain N-acetylaspartate, creatine, and choline levels also decrease as maternal stress score increases [4]. The same group also suggests positive associations between maternal stress and anxiety and lactate levels in the fetal brain [103]. Metabolic alterations in the in utero fetal brain have been shown to precede morphologic brain changes [104] and may provide new insights into the mechanisms that underlie impairments to fetal brain development concerning prenatal maternal psychological distress [105]. These data suggest that altered brain metabolism in the setting of maternal psychological distress may have important implications for impaired brain structural and functional development in the offspring.

#### Prenatal maternal psychological distress and functional brain connectivity

Prenatal exposure to psychological distress is also associated with altered functional connectivity [19, 37]. In healthy fetuses, in utero exposure to heightened maternal anxiety is linked to altered functional connectivity in sensorimotor and association cortices. Connections that develop earlier (i.e., brainstem and sensorimotor areas) are stronger in high-anxiety states, while parieto-frontal and occipital connections that develop later are weaker. Increased hippocampal connectivity to medial and superior frontal gyri is also present in fetuses of women with high trait anxiety [33]. Higher maternal negative affect and stress are linked to alterations in the insula and inferior cerebellar functional connectivity as well as increased sleep problems at 3–5 years old, although connectivity changes do not seem to mediate the maternal stress-behavior relationship [106]. Recently, increases in hippocampal connectivity due to elevated maternal stress and cortisol have also been reported [107]. Increased connectivity to the right posterior parietal cortex is associated with elevated maternal stress while increased coupling with the medial prefrontal area and dorsal anterior cingulate cortex is related to increased maternal cortisol. Importantly, the latter association, but not the former, is moderated by fetal sex. This suggests that there are different mechanisms by which stress and cortisol impact the developing hippocampal circuitry. Altogether, these studies demonstrate the susceptibility of fetal neural circuitry, particularly the limbic structures, to maternal psychological distress.

Aberrant hippocampal connectivity is also reported in infants with prenatal exposure to elevated maternal distress. Symptoms of stress correlate inversely with connectivity to the dorsal and mid-cingulate areas, but positively to the temporal lobe; most notably, increased 2nd-trimester cortisol levels correlate with alterations in hippocampal connectivity [108]. Similarly, the amygdala and medial prefrontal cortex coactivate less in stress-exposed newborns. This contrasts with structural integrity, which increases between these regions [109]. In-utero exposure to maternal stress also exacerbates weakened limbic connectivity in very premature newborns, such that reductions in connectivity between the amygdala and subcortical areas are greater in stress-exposed preterm infants compared to non-exposed preterm infants [19]. Interestingly, weaker connectivity between the amygdala and anterior default mode network is observed in newborns whose mothers experience high psychosocial stress and are living in neighborhoods with high property or violent crime rates. The brain-neighborhood association is mediated, in part, by maternal psychosocial stress. Weakened newborn amygdala-hippocampus connectivity is also related to violent crime [110]. Alterations in infant amygdala circuitry are also reported in cases of maternal depression. Experiencing elevated symptoms of maternal depression during the 2^nd^ trimester is closely associated with increased connectivity of the amygdala to the left temporal cortex, insula, anterior cingulate, and the medial and ventromedial prefrontal cortices. Notably, these areas are involved in socio-emotional processing and memory, similar to regions implicated in depression in adults [37]. Later exposure (i.e., 3rd trimester) to heightened depression symptoms is linked to decreased connectivity to prefrontal circuits at around 5 weeks of life [111]. Higher maternal depression scores also correlate with weaker connectivity between bilateral hippocampi and posterior cingulate cortex in newborns [108]. Associations between the amplitude of regional neuronal activity (i.e., the fractional amplitude of low-frequency fluctuation, as opposed to inter-regional co-activation revealed by the canonical BOLD) in newborn’s medial prefrontal cortex and combined maternal depression and anxiety scores have also been reported [112]. Infants of women who were pregnant during the COVID-19 pandemic with low social support reportedly display weaker connectivity between the right amygdala and superior orbitofrontal cortex [113].

Associations between exposure to maternal psychological distress and connectivity persist beyond the perinatal period. In young girls, there is an association between greater maternal depression and weakened connectivity of the amygdala to the cortico-striatal circuitry, particularly in the insula, putamen, orbitofrontal cortex, and temporal pole [114]. Similarly, elevated maternal anxiety during the 2nd trimester is also linked to greater negative amygdala connectivity to bilateral somatosensory cortices and the left inferior parietal lobule [115]. In another study, exposure to maternal depression in utero is linked to amygdala hyperresponsivity during childhood [116]. Adult offspring of pregnant women with high anxiety display weakened connectivity between the medial prefrontal cortex and inferior gyrus and between the left lateral prefrontal cortex and sensorimotor cortex. In women exposed to high levels of prenatal stress, the stress and functional connectivity between the left medial temporal lobe and the subgenual anterior cingulate cortex are highly correlated [28]. More importantly, orbitofrontal cortex and middle temporal cortex connectivity track the severity of depression symptoms. Altogether, functional connectivity findings suggest that disrupted neural circuitry related to maternal psychological distress begins early and persists throughout the lifespan and underscore the importance of addressing maternal mental health issues to improve maternal-fetal care.

#### Sex differences in brain development after prenatal stress exposure

There is a body of literature which suggests alterations in brain development due to maternal stress, anxiety, or depression during pregnancy may be sex-specific. Studies suggest that maternal depression measured at 26 GW and saliva cortisol levels at 15 GW are associated with larger right amygdala volume in girls only [26, 39]. Also, elevated pregnancy-related anxiety in the 2nd trimester is related to greater left-relative amygdala volume in girls vs. boys [22]. These results underscore the selective vulnerability of the amygdala to prenatal maternal stress, especially in girls [39]. Additionally, early prenatal maternal stress has been associated with increased temporal cortical gyrification index in female adults [30]. Similarly, sexually dimorphic functional brain changes that are related to stress have been documented in the past. Sex-specific associations between maternal cortisol and amygdala connectivity in newborns has also been demonstrated. In females, higher cortisol levels are correlated with greater amygdala connectivity to diverse networks (e.g., default mode network and emotion regulation); the reverse is true in males [117]. Elucidating sexual dimorphism in brain changes related to maternal psychological distress is critical for understanding the complex interplay between genetics, prenatal environment, and neurodevelopment. It underscores the importance of considering sex as an important variable while studying the effects of maternal mental health on offspring brain development.

### Neurobehavioral outcomes in the offspring of pregnancies complicated by elevated maternal psychological distress

Prenatal maternal psychological distress has been shown to have enduring consequences on long-term neurobehavioral development in the offspring [13, 24, 26, 34, 40–46], partially through altered brain structure and circuitry [118, 119]. Prenatal maternal stress has recently been associated with decreased cognitive performance of toddlers at 18 months [34]. This association is partially mediated by fetal left hippocampal volume [34]. At later ages, prenatal maternal depression and disaster-related stress are associated with externalizing behaviors in children. These associations are mediated by child cortical thinning in prefrontal areas of the right hemisphere [24], amygdala volume [88], and an altered structural connectivity between the amygdala and frontal cortex [95]. In addition, elevated levels of maternal pregnancy-specific anxiety are also associated with child executive function, including lower inhibitory control in girls and lower visuospatial working memory performance in both boys and girls [44]. Moreover, a large body of research shows that prenatal maternal psychological distress is associated with mental health problems in children, adolescents, and even adult offspring [42, 45, 85]. One study suggests that elevated pregnancy-related anxiety is associated with more emotional symptoms, peer relationship problems, and overall child difficulties in young children. The child left amygdala volume may partly mediate the associations between maternal anxiety and child behavioral difficulties [22]. The amygdala volume is also suggested to partially mediate the associations between elevated maternal cortisol levels at 15 GW and affective problems in girls [26]. In addition, prenatal maternal stress has been associated with elevated depressive symptoms in adolescent offspring, and early childhood changes in fronto-temporal cortical thickness in the setting of prenatal maternal stress are correlated with adolescent depressive symptoms [85]. In adult offspring, prenatal maternal stress and depression are linked to increased cortical gyrification index in the temporal region and the brain age gap (i.e., the differences between chronological and structural brain age). These brain changes are further related to adult mood disturbances [29, 30].

Additionally, some of the reported functional alterations related to prenatal exposure to maternal psychological distress have been linked to neurobehavioral outcomes. One previous study shows that maternal cortisol predicts internalizing score on the Child Behavior Checklist at 2 years of age. In girls, this relationship is mediated by increased amygdala connectivity [117]. Also, connectivity between the hippocampus and dorsal anterior cingulate cortex, which is inversely associated with maternal stress, has been noted to correlate positively with infant memory [108]. Low socio-economic status, which has been linked to maternal stress [120, 121], has also been correlated with altered striatal and medial prefrontal connectivity at birth, which mediates the relationship between low socio-economic status and behavioral inhibition at 2 years of age [122].

These studies suggest that prenatal maternal mental distress, even if not reaching the severity of a mental disorder, has an impact on neurodevelopmental outcomes in the offspring, and cannot be ignored.

### Mechanistic underpinnings of brain development changes in stress response

**Fig. 3:**
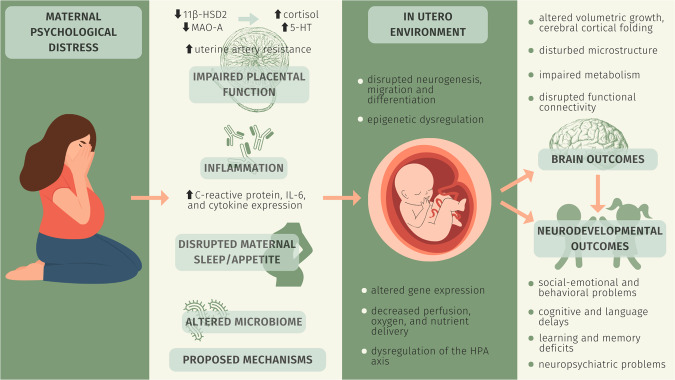
Prenatal maternal distress and outcomes. Brain and neurobehavior developmental outcomes of prenatal maternal psychological distress and possible mechanisms.

It is well-known that the intra-uterine environment plays a critical role in supporting fetal brain growth and development. The human brain begins to develop at the embryonic stage and continues to grow rapidly throughout the fetal stage, particularly over the third trimester of pregnancy [50]. Notably, this rapid period of fetal brain growth and maturation is sensitive to hostile intra-uterine conditions, such as prenatal malnutrition [123], infection [124], drugs [125], and stress [126]. The mechanisms by which maternal psychological distress influences early brain development are complex and multifactorial. Impaired placental function has previously been implicated, including a decrease in placental expression of monoamine oxidase A [127] and 11β-hydroxysteroid dehydrogenase type 2 [128], which may increase fetal exposure to 5-hydroxytryptamine and cortisol, respectively. 5-hydroxytryptamine affects cell neurogenesis, migration, and differentiation of the fetal brain [129], and elevated cortisol exposure affects gene expression in fetal brain cells [130]. In addition, maternal distress is associated with increased uterine artery resistance, which may impair placental perfusion and decrease oxygen and nutrient delivery to the fetal brain [131]. A recent study also suggests that elevated prenatal maternal depression is associated with decreased fetal middle cerebral arterial resistive index, which reflects a redistribution of the combined fetal cardiac output to the brain [35]. Elevated prenatal maternal stress is also suggested to alter the microbiome, and the maternal microbiome has been associated with the development of the fetal brain and infant microbiome [132–134]. Disrupted maternal sleep and appetite under stress is another possible factor [135]. Moreover, maternal inflammation may play a role, given that maternal stress has been associated with increased inflammatory markers and altered cytokine production during pregnancy [136–139]. The literature points to a relationship between maternal Interleukin-6 concentration during pregnancy and altered newborn brain structure and functional connectivity [140, 141]. Additionally, C-reactive protein (CRP), an inflammatory marker, is elevated as prenatal maternal mental distress increases [142, 143], and elevated gestational CRP levels have been associated with increased risk of preterm birth [144], adverse infant and child brain developmental outcomes [144, 145], as well as autism and schizophrenia in the offspring [146, 147]. The hypothalamic pituitary adrenal (HPA) axis also plays a central role in mediating the effect of maternal psychological distress on the fetal brain [148]. Interestingly, there are reports that maternal psychological distress affects DNA methylation in the corticotropin-releasing hormone and glucocorticoid receptor gene (*NR3C1*) in neonatal cord blood [149], and brain-derived neurotrophic factor in infants [150]. Additionally, there are reports of higher stress-related gene *SLC6A4* methylation in newborns after exposure to elevated prenatal stress. The *SLC6A4* methylation is suggested to influence infants’ temperament [151]. These studies address potential disturbances in fetal epigenetic regulation. Importantly, the literature suggests a range of prenatal exposures that can collectively impact fetal development [152–156]. Some factors frequently overlap and may trigger similar biological pathways [152, 153]. In addition to psychological distress, exposures that may impact fetal brain development include social determinants of health (income, education, racism, health care access/quality, neighborhood disadvantage, parental care, social support), lifestyle factors (smoking, diet, sleep, exercise, alcohol intake), physical and chemical exposures (radiation, pesticides, food and water contaminants, air pollution, substance use), medical problems (infections, hypertension, diabetes, obesity, malnutrition, chronic medical conditions), ecosystems and climate (green space, population density), etc. These factors can be associated with one another [152]. It is possible that the association between distress and the physiological response may be mediated by other variables, or distress may be the mediating variable to other exposures. The shared biological mechanisms make it difficult to precisely map prenatal exposures to their effects on fetal brain development, highlighting the need to study these factors as a group rather than as single entities. Lastly, it is noteworthy that maternal psychological stress during pregnancy may not be transient but persistent across the postnatal period with subsequent influences on both parent-child interactions and infant self-regulation [35]. High levels of maternal psychological distress during the postnatal period may increase the possibility of exposing children to a harsh parenting environment which could have lasting detrimental impacts on children while increasing the likelihood of internalizing and externalizing problems in the short and long term [157]. The possible mechanisms underpinning brain development changes due to stress response are summarized in Fig. 3.

### Current treatment for elevated maternal psychological distress

Maternal psychological distress is prevalent during pregnancy. The main treatment strategies include pharmacotherapy and psychotherapy.

#### Pharmacology

Although there are many antidepressants available, medication choices are often more limited for pregnant women. Selective serotonin reuptake inhibitors (SSRIs) and serotonin and norepinephrine reuptake inhibitors (SNRIs) are the most commonly used antidepressants during pregnancy and the postpartum period. SSRIs work by increasing the levels of serotonin in the brain. SNRIs work similarly to SSRIs by increasing the levels of serotonin and norepinephrine in the brain. Both are considered safe for use in pregnancy [158, 159]; however, they still pose some risks. A treatment of SSRIs/SNRIs in the third trimester of pregnancy may result in increased incidences of neonatal adaptation syndrome, which is characterized by a low Apgar score, hypoglycemia, weak muscle tone, respiratory difficulties, and total restlessness [160, 161]. While the adaptation syndrome is considered to be temporary, newborns exposed to SSRIs or SNRIs at the end of the pregnancy could require longer hospitalization, tube feeding, and breathing support [161]. The current literature also suggests that women who received SSRI treatment during pregnancy have a significantly higher risk of developing preterm birth compared with controls and depressed women not on SSRIs [162]. Prenatal SSRI exposure is linked with alterations in the postnatal brain, including increased gray matter volume in the right amygdala and right insula, as well as increased structural connectivity between the right amygdala and right insula in infants [129]. It also relates to higher connectivity in putative auditory resting-state networks [163] and lower fractional anisotropy, increased mean and radial diffusivity for multiple white matter fiber bundles in newborns [164]. Children exposed to prenatal SSRIs are also more likely to have Chiari I malformations when compared to children with no SSRI exposure [165]. Additionally, a meta-analysis study suggests that SSRI use during pregnancy may have long-term effects on neurobehavior and performance in the offspring [166]. Infants and toddlers exposed to SSRIs prenatally have lower motor development scores and decreased motor control [167]. In addition, infants who are exposed to SSRIs may have an attenuated pain response and an abnormal EEG, which is suggestive of encephalopathy. This attenuated response may result from increased serotonin (5-HT) and GABA agonists in the fetal brain under SSRI exposure [166, 168]. In addition to SSRIs and SNRIs, tricyclic antidepressants have also been prescribed to pregnant women for several decades. However, tricyclic antidepressants are considered to cause more side effects than SSRIs and SNRIs [169].

#### Non-pharmacology

Psychotherapy is an effective and medication-free way of managing and treating mental distress. Psychological interventions include different treatment formats (i.e., individual therapy, group therapy, or guided self-help) [170, 171]. There are many types of psychotherapy available, including but not limited to cognitive-behavior therapy (CBT), interpersonal psychotherapy (IPT), supportive treatment (ST), psychodynamic treatment (PDT), mindfulness-based interventions, and behavioral activation therapy [172, 173]. A review study suggests that for the treatment of depression, patients receiving CBT are more likely to see improvements than those receiving PDT, IPT, ST, or treatment as usual [174]. For addressing prenatal psychological distress, CBT helps to identify and change negative thinking and behavioral patterns that affect how the patients feel. CBT is considered an acceptable, feasible, and effective intervention for women with anxiety and depression during pregnancy [175, 176]. IPT, which focuses on improving the patients’ relationships with others, is also commonly recommended during pregnancy. IPT shows a moderate treatment effect for prenatal anxiety and depression [175]. Mindfulness-based interventions can be effective in improving prenatal maternal anxiety and depressive symptoms [177, 178]. In addition, body-oriented interventions and acupuncture may also reduce prenatal depressive symptoms [175]. A review study of Black and Latin American women in the United States concludes that participants with psychotherapy interventions, including CBT (applied in most studies), IPT, acceptance and commitment therapy, problem-solving therapy, CBT plus positive parenting, Enhanced Triple P for Baby and Mellow Bumps, Motherly app plus brief online CBT, all showed less prenatal and postpartum anxiety than those in the routine care-review paper [179]. Psychotherapy has also been suggested as an effective way of reducing postpartum depression symptoms and improving coping with stress and negative emotions in depressed mothers [180–182], as well as improving the patterns of interactions between mothers and their children [157, 182]. Importantly, these studies find that psychotherapy in distressed parents has a positive impact on the mental health of their children [182–184]. Results show children of families receiving cognitive and behavioral-based interventions demonstrate fewer severe anxiety symptoms overall and have a significantly lower onset rate for anxiety disorders compared to those assigned to the control group over a 1-year follow-up period [183, 184]. Other medication-free options that may help improve maternal psychological distress symptoms include music therapy [185], journal therapy [186], light therapy [187], hypnosis [188], yoga exercise [189], omega-3 fatty acid supplementation [187], and getting enough quality sleep [190].

To compare the effectiveness of psychological and pharmacological treatments, a review paper that covers 30 randomized controlled trials of 3178 participants from North America, Mexico, and the United Kingdom suggests that treatment for depression with SSRIs is more effective than psychological therapy and the effect of treatment with other antidepressants is similar to that of psychological therapy. In the short-term treatment of depression, psychological and pharmacological therapies have similar efficacy [171]. Another meta-analysis study also concludes that the efficacy of psychotherapy for mild to moderate depression is about the same as the efficacy of pharmacotherapy, and that combined treatment is more effective than psychotherapy alone or pharmacotherapy alone [191]. Drop-out rates are suggested to be lower in psychological therapy as compared to pharmacological therapy [171].

### Future directions

Even though maternal psychological distress is the most common complication during pregnancy and the postpartum period, up to 70% of women impacted remain undiagnosed and thus untreated. Among the women who receive screening, only one-third with depression receive formal mental health care [192]. These findings highlight the need for routine mental health surveillance for all women during pregnancy and postpartum. In addition to universal screening, targeted psychological interventions are recommended as the most effective approach to prevent prenatal and postnatal depression, especially among those with risk factors, such as a history of mental disorders, financial concerns, unwanted pregnancies, and a lack of support [193, 194]. Studies suggest that universal prevention (e.g., CBT, IPT, mindfulness, and psychoeducation) during pregnancy is effective in decreasing symptoms of maternal distress compared to routine care and recommends psychotherapy as a part of standard prenatal self-care [178, 194, 195]. Preventive mental health care during pregnancy should complement usual prenatal care to improve symptoms of maternal depression and anxiety [178, 194, 195]. There is also a desire to personalize interventions and treatments to fit each patient’s needs. Social support, which includes support in developing and maintaining personal, family, and social relationships, may also be a vital protective factor for mental health across demographics [196–199].

Advances in quantitative MRI have provided a unique window to study the fetal brain and greatly improved our understanding of the role of maternal psychological distress on fetal neurodevelopment. Imaging has provided previously unavailable clues on possible neurobiological substrates for behavioral phenotypes later seen in children exposed to symptoms of stress, anxiety, and depression in utero. The convergence of brain imaging findings on susceptible brain structures such as the amygdala, hippocampus, and medial frontal cortical areas, regions previously implicated in the stress response, suggests potential mechanisms by which maternal stress is relayed to the developing fetuses. Further progress in the field will require large-scale, longitudinal studies that leverage structural and functional MRI modalities to advance our understanding of how maternal mood impacts the developing brain. By collaboratively building large databases that capture serial measures of brain development at key developmental intervals (prenatal, neonatal, infant, toddler, school-age, adolescents), researchers and clinicians can formulate more robust and generalizable brain-behavior models, but also probe individual variations in the maternal-fetal stress response. Identifying in utero brain biomarkers that reliably predict long-term outcomes will rely heavily on the development of precision fetal imaging to support more timely and accurate neurologic surveillance and targeted early interventions to measure treatment response [200]. To complement precision fetal brain imaging, a multidimensional framework that incorporates genetics, epigenetics, computational neuroscience, neuropsychology, and medicine is urgently needed to characterize the complex interplay between the developing fetus and the external environment, particularly for interrogating the mechanisms underlying intergenerational transmission of stress.
